# First Report of *Microcystis* Strains Producing MC-FR and -WR Toxins in Japan

**DOI:** 10.3390/toxins11090521

**Published:** 2019-09-09

**Authors:** Tsuyoshi Ikehara, Kyoko Kuniyoshi, Haruyo Yamaguchi, Yuuhiko Tanabe, Tomoharu Sano, Masahiro Yoshimoto, Naomasa Oshiro, Shihoko Nakashima, Mina Yasumoto-Hirose

**Affiliations:** 1 Department of Food Science and Technology, National Fisheries University, 2-7-1 Nagata-honmachi, Shimonoseki, Yamaguchi 759-6595, Japan; 2 National Institute of Health Sciences, 3-25-26 Tonomachi, Kawasaki, Kanagawa 210-9501, Japan (K.K.) (N.O.); 3 National Institute for Environmental Studies, Onogawa 16-2, Tsukuba, Ibaraki 305-8506, Japan (H.Y.) (Y.T.) (T.S.); 4 Okinawa Institute for the Conservation of the Environment Co. Ltd., 7-11 Suzaki, Uruma, Okinawa 904-2234, Japan; 5 Faculty of Sports and Health Science, Fukuoka University, Fukuoka 814-0180, Japan; 6 Tropical Technology Plus Ltd., 5-1 Suzaki, Uruma, Okinawa 904-2234, Japan

**Keywords:** Cyanobacteria, *Microcystis*, microcystins, environmental water, liquid chromatography–mass spectrometry, protein phosphatase 2A inhibition assay

## Abstract

Microcystins (MCs) are a group of cyclic heptapeptide hepatotoxins produced by *Microcystis* and several other genera of cyanobacteria. Many structural variants have been characterized using various methods such as liquid chromatography–mass spectrometry (LC-MS) analysis, enzyme-linked immunosorbent assay (ELISA) and protein phosphatase 2A (PP2A) inhibition assay. The representative MC, MC-LR, and related cyanobacterial toxins strongly inhibit PP2A activity and can therefore be assayed by measuring the extent of PP2A inhibition. However, these methods require reference toxin standards for the quantification and identification of known MCs. To obtain various MC-producing cyanobacterial strains, we surveyed and collected MC-producing cyanobacteria from environmental sources of water in Okinawa, Japan. Using a dual assay (LC-MS analysis and PP2A inhibition assay), we identified and isolated *Microcystis* strains producing five MC variants (MC-LR, -RR, -LA, -FR and -WR). Approximately 4 mg of MC-WR and -FR toxins were purified from the laboratory culture of the *Microcystis* isolate NIES-4344. Pure MC-WR and -FR variants were prepared for future use as toxin standards in LC-MS analysis. Phylogenetic analysis based on *ftsZ* revealed that the NIES-4344 strain belongs to the identified groups in *Microcystis aeruginosa*. This is the first report of *Microcystis* strains producing mainly MC-WR and -FR toxins in Japan.

## 1. Introduction

Worldwide blooms of toxic cyanobacteria (blue-green algae) commonly occur in fresh, brackish and marine waters. Microcystins (MCs) are a group of cyclic heptapeptide hepatotoxins produced by *Microcystis* and several other genera of cyanobacteria. MCs are composed of five common amino acids plus a pair of variable L-amino acids. More than 250 structural variants of MCs have been characterized in bloom samples and cultured strains of cyanobacteria to date [1,2,3]. The structural variation in MCs is based on either the two variable amino acids or chemical modifications around the molecules. All MCs are highly toxic to many life forms, including aquatic organisms, wildlife, livestock and humans, and MC-LR, the representative MC variant, is the most toxic. In 1996, MC caused the death of over 50 patients on hemodialysis in Caruaru, Brazil [4,5]. Given the toxicity of MCs, the World Health Organization (WHO) has recommended a maximum allowable level of 1 μg/L of MC-LR or its equivalent in water [6]. To monitor the level of MCs in aquatic environments, a powerful analytical tool is needed. High-performance liquid chromatography (HPLC) and liquid chromatography–mass spectrometry (LC-MS) analyses are used for quantification and identification of known MCs, while protein phosphatase 2A (PP2A) inhibition assay and enzyme-linked immunosorbent assay (ELISA) are used for the rapid detection and quantification of total MCs. Recently, fast detection strategies for cyanobacterial blooms and associated cyanotoxins were reported [7,8]. These methods require reference toxin standards for the quantification and identification of known MCs. However, commercially available MC standards are limited to a few major MC variants, such as MC-LR and MC-RR. Therefore, a stable supply of diverse MC standards is crucial for MC analysis.

MCs are known to inhibit the activity of serine/threonine protein phosphatases (PPs) such as PP1 and PP2A [9,10]. We produced a recombinant catalytic subunit of human PP2A (rhPP2Ac) via genetic engineering using the baculovirus expression system with High Five insect cells [11,12]. The highly purified rhPP2Ac was biologically active and was inhibited by MC variants [13], okadaic acid and okadaic acid analogs (OAs). Previous studies have demonstrated the suitability of rhPP2Ac in the PP2A inhibition assay, for example, for the detection of OAs in shellfish [14,15], and detection and quantification of MCs in environmental water resources [16].

Here, we report the toxin composition of *Microcystis* strains producing MCs isolated from environmental water resources in Okinawa locating at subtropical region in Japan. Five MC variants were identified including MC-RR, -LR, -YR, -WR, -FR and -LA. *Microcystis* strains (NIES-4344 and NIES-4345) producing both MC-WR and MC-FR were isolated, and the two MC variants were purified from the laboratory culture of the isolates (NIES-4344). Pure MC-WR and MC-FR were prepared for use as reference materials in LC-MS analysis. Multilocus sequence typing (MLST) with seven selected housekeeping loci (*ftsZ*, *glnA*, *gltX*, *gyrB*, *pgi*, *recA* and *tpi*) has revealed that *Microcystis aeruginosa* is divided into several groups [17,18,19]. In this study, phylogenetic analysis based on *ftsZ*, one of the seven loci, revealed that the NIES-4344 strain belongs to group X, while NIES-4345 belongs to none of the identified groups. *Microcystis* strains previously identified in Japan have been reported to produce mainly MC-RR (50%), MC-LR (30%) and MC-YR (14%) variants [20]. Thus, this is the first report of strains producing MC-WR and -FR in Japan.

## 2. Results

**Figure 1:**
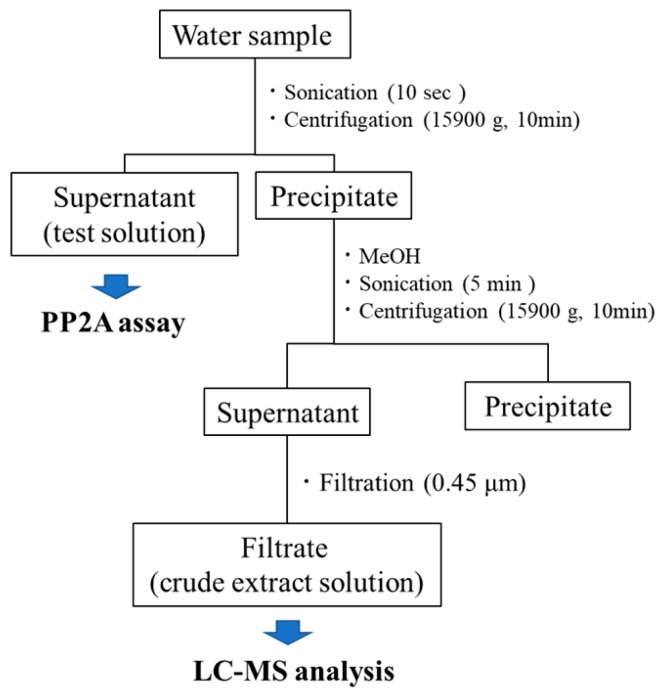
Preparation of test solutions for PP2A inhibition assay and LC-MS analysis.

Cyanobacterial water blooms were identified in 13 out of 98 sites surveyed at Okinawa prefecture in Japan (Supplemental Figure S1). To detect and identify the MCs in these water blooms, surface water containing cyanobacteria was collected from the 13 sites and used to prepare test solutions, which were then analyzed by the PP2A assay and LC-MS analysis (Figure 1). To determine the presence of MCs, the PP2A activity was measured in the test solutions (Table 1).

**Figure 2:**
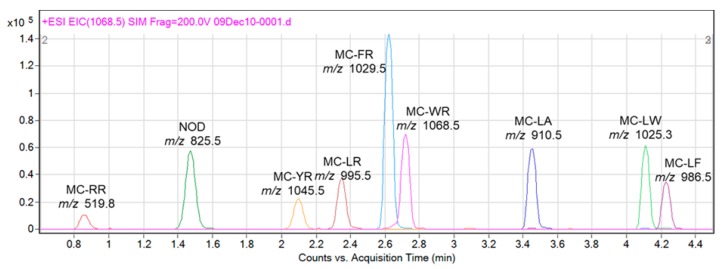
Chromatograms of nine standard MCs obtained via LC-MS analysis.

**Figure 3:**
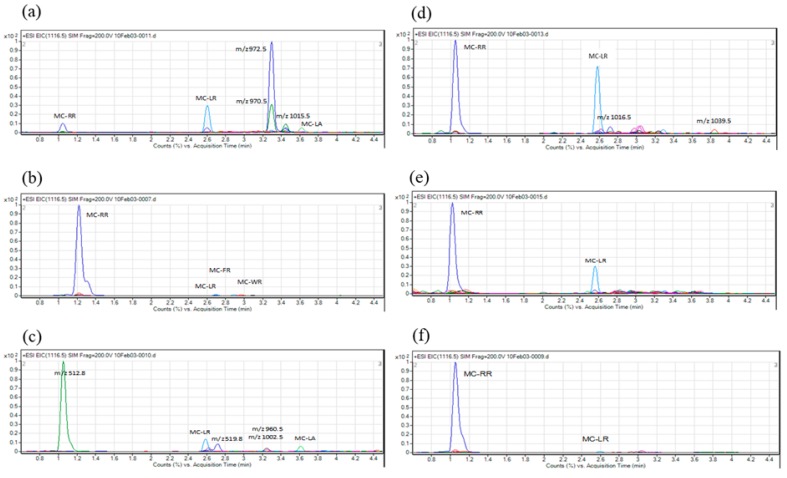
Chromatograms showing the composition of MCs isolated from crude extracts prepared from water samples containing cyanobacteria at six sites. (**a**) Site No. 1; (**b**) Site No. 7; (**c**) Site No. 8; (**d**) Site No. 10; (**e**) Site No. 11; (**f**) Site No. 12.

**Figure 4:**
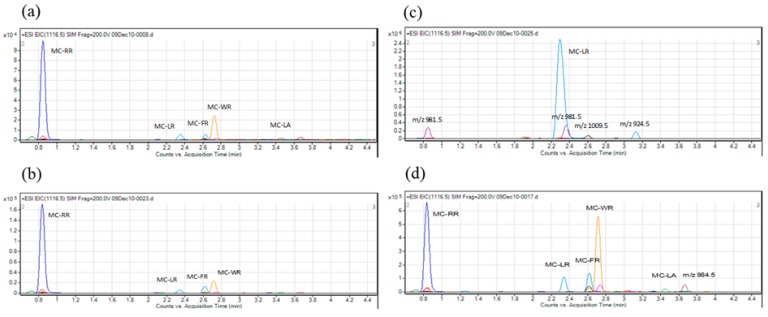
Chromatograms showing the composition of MCs of cyanobacterial strains isolated from different sampling sites. (**a**) Site No. 1; (**b**,**c**) Site No. 7; (**d**) Site No. 12.

The MC inhibitory activity against PP2A of samples collected from six sites (site No. 1, 7, 8, 10, 11 and 12) varied from 43.3% to 82.4%, whereas that of samples collected from the other seven sites (site No. 2, 3, 4, 5, 6, 9 and 13) was below 10% (range: 3.4% to 9.5%). These results indicated the possibility that cyanobacteria collected from six sites produced MCs. PP2A activity rate and calibration curve using net absorbance value obtained by the PP2A assay with MC-LR standard solution was shown in Supplemental Figure S2 as reference data. To determine the composition of MCs produced by these cyanobacteria, we conducted LC-MS analysis of the crude extract prepared from the cyanobacteria-containing water samples using nine reference toxins (Figure 2). Five MC variants (MC-LR, -RR, -LA, -FR and -WR) were detected in the crude extracts (Table 1, Figure 3). MC-LR was detected at all six sites (Figure 3a–f), MC-RR at five sites (Figure 3c), MC-LA at two sites (Figure 3a,c) and MC-FR and MC-WR only at one site each (Figure 3b). Additionally, unialgal strains were isolated from the same water samples (Table 1), and the representative chromatographs of the isolates are shown in Figure 4. Five MC variants (MC-RR, -LR, -FR, -WR and -LA) were detected in the isolates (Figure 4a,d). The two isolates shown in Figure 4b and 4c were derived from same sampling site (site No. 7; Table 1). One isolate mainly produced MC-RR (Figure 4b), and the other isolated mainly produced MC-LR (Figure 4c). These results indicate that the cyanobacterial water bloom consists of several MC-producing strains, and each strain produces one or more MC variants.

**Figure 5:**
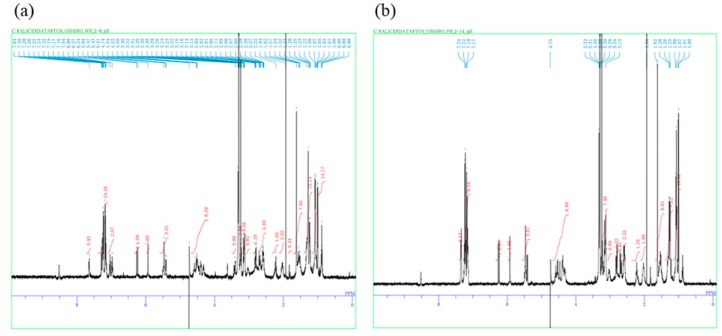
^1^H NMR spectra of purified MC variants. (**a**) MC-WR; (**b**) MC-FR.

Furthermore, two *Microcystis* unialgal strains (NIES-4344 and NIES-4345) producing MC-WR and MC-FR variants were isolated from Okinawa’s artificial ponds (Site No. 14 and 15 shown in Supplemental Figure S1b), and approximately 4 mg of each variant was purified from 490 g (wet weight) of the laboratory culture of the NIES-4344 isolate. The purity of these MC variants was confirmed by ^l^H nuclear magnetic resonance (NMR) spectrometry (Figure 5). In addition to microscopic observation of the distinguishing morphological characters of *Microcystis* strains, sequencing of the *fts*Z gene was performed to identify the intraspecific group among *Microcystis aeruginosa*. Sequence data of the *fts*Z gene have been deposited at the DNA Data Bank of Japan (DDBJ) under the accession numbers LC495738 and LC495739. Phylogenetic analysis of the *fts*Z gene sequences revealed that the NIES-4344 strain belongs to group X, whereas NIES-4345 belongs to none of the identified groups previously reported [19] (Figure S3).

## 3. Discussion

Humans and animals are at risk of exposure to MCs present in drinking water or in foods. Therefore, the development of good analytical methods for the detection of MCs is important for food safety and security and public health. These methods require reference toxin standards for quantification and identification of known MCs. Although many commercial MC standards are available for analysis such as MC-LR, -RR, -YR, -LA, -LF and -LW, securing toxin standards stably is important. In this study, several MC variants were detected in surface water containing cyanobacteria in Okinawa using a dual assay, comprising LC-MS analysis and PP2A inhibition assay, with the rhPP2Ac. The major variants identified in Okinawa were MC-LR and -RR. In addition to these dominant variants, MC-WR and -FR were detected as minor variants. MC-LR, -RR and -YR have been reported as the major toxins in bloom samples in Japan [20], and MC-WR and -FR have been reported in Japan for the first time. The Okinawa prefecture is located at the far south end of Japan. It would be interesting to know if the growth conditions of cyanobacteria are responsible for the different MC variants. To test the possibility, it would be necessary to reveal toxin profile of cyanobacteria at various areas across the world. Although the MC-WR and -FR variants have been detected at or isolated as field samples from a variety of fresh water sources in the world such as USA (Illinois [21] and San Francisco [22]), Portugal [23], Taiwan [24], India [25], Morocco [26], Berlin [27] and New Zealand [28], only a few commercial MC-WR and MC-FR variants have become available to date. In this study, we prepared pure MC-WR and -FR variants for future use in LC-MS analysis as reference toxin standards. Moreover, a stable supply of MC-WR and -FR variants will be available by the culture of strains producing these MCs. Nonetheless, a stable supply of other MC variants is also needed. Previously, the inhibitory potency of 21 MC variants was determined in a PP2A inhibition assay using the rhPP2Ac, and the conversion factor, which enables the precise conversion of the amount of various MCs to MC-LR equivalents, was detected using instrumental methods [13]. This suggests that rhPP2Ac will be highly useful in future studies for the detection and quantification of MC variants as an effective and rapid method to identify various MC-producing cyanobacteria. The combination of the PP2A inhibition assay with LC-MS analysis constitutes an efficient monitoring procedure for the rapid assessment of environmental and health risk associated with the proliferation of hepatotoxic cyanobacteria [29]. The purified MC-WR and MC-FR will be useful as toxin standards for the LC-MS method in future monitoring studies. Because the isolated strain NIES-4345 did not belong to any of the known groups, according to the *ftsZ* gene analysis, further analyses of additional genes via MLST will be necessary for the determination of groups. We will continue the survey of toxin composition and phylogenetic analysis on MC-producing cyanobacteria strains from environmental sources of water, it would help clarify the relationship of toxin profile and genetic reason in MC-producing cyanobacteria strains and prepare the MC toxin standards for LC/MS analysis.

## 4. Materials and Methods

### 4.1. Water Sample Collection

To identify MC-producing Microcystis strains, surface water containing cyanobacterial water bloom were collected from 13 sites (Supplemental Figure S1) such as reservoirs and artificial ponds in Okinawa prefecture, Japan.

### 4.2. Sample Preparation

Test solutions for the PP2A assay and LC-MS analysis were prepared from water samples collected from the 13 sites. One liter of water sample from each site was sonicated for 10 s to remove aerial substances surrounding cells and then centrifuged at 15,900× *g* for 10 min. The supernatant (test solution) was used for the PP2A assay. The precipitate was added to an equivalent volume of methanol (MeOH) and sonicated for 5 min to extract intracellular MCs. The suspension was centrifuged at 15,900× *g* for 10 min, and the supernatant was filtered. The filtrate (crude extract solution) was diluted approximately 20-fold using ultra-pure water to reduce the MeOH content. The resulting sample was used for LC-MS analysis.

### 4.3. PP2A Inhibition Assay

The rhPP2A was synthesized in insect cells (High Five; Invitrogen, Carlsbad, CA, USA) by the infection of recombinant baculovirus encoding His_x8_-tagged human PP2Acα using a baculovirus expression system and purified, as described previously [11,12]. The PP2A inhibition assay using rhPP2Ac was performed in 96-well plates, as described previously [16]. Each well contained 50 µL of the test solution and 100 µL of *p*-nitrophenylphosphate (*p*-NPP; 3 mM final concentration). The reaction was started by adding 100 µL (0.08 units) of the rhPP2A and continued for 30 min at 36 °C. The hydrolysis of *p*-NPP to *p*-nitrophenol (*p*-NP) was recorded using a microplate reader at 405 nm against 492 nm as a reference in triplicate. Ultra-pure water was used as a negative control. The MC inhibitory activity against PP2A (%) was calculated using the following equation:

MC inhibitory activity against PP2A (%) = (1 − A_T_/A_C_) × 100 (1)

where A_T_ is the absorbance of test solution, and A_C_ is the absorbance of the negative control.

### 4.4. MC Standards

MC-RR and -LR standard solutions were obtained from Kanto Chemical Co., Inc. (Tokyo, Japan). MC-YR was obtained from Wako (Osaka, Japan). MC-LW and -LF were obtained from Alexis Corporation (San Diego, CA, USA), and nodularin was purchased from Calbiochem (San Diego, CA, USA). MC-FR and -WR were isolated from cultured strains and bloom samples, as reported previously [30], and a mixture of these MCs was used as the standard for LC-MS. 

### 4.5. LC-MS Analysis

The LC/ESI/MS system consisted of a 6460 Triple Quad MS and 1200 Series systems (Agilent, Santa Clara, CA, USA). HPLC separations were performed on Eclipse XDB-C18 (50 × 2.l mm; 1.8 µm; Agilent, Santa Clara, CA, USA). Water was used as eluent A, and acetonitrile:water solution (95:5, *v*/*v*) was used as eluent B; both contained 2 mM ammonium formate and 50 mM formic acid [31]. The flow rate was 0.6 mL/min. MCs were separated using a gradient elution program: eluent B gradient from 30% to 70% for 4 min followed by hold on 70% for 0.5 min. The following parameters were used for MS: ionization, ESI; ion mode, positive; nebulizer gas, N_2_ 35 psi.; capillary voltage. 5000 V; dry gas, 10 L/min of N_2_ at 300 °C. Respective ions of [M+H]^+^ or [M+2H]^2+^ of MCs were monitored by selected ion monitoring (SIM) mode.

### 4.6. Isolation and Culture of Cyanobacterial Strains

Cyanobacterial genera and species were identified by microscopic observation of the distinguishing morphological characteristics, as described previously [32]. Colonies of cyanobacteria collected from natural water samples were picked under a stereoscopic microscope using a capillary pipette, and cells identified as *Microcystis* were inoculated in a 24-well plate filled with 1 mL of TS-15 medium [33]. The isolated cells were incubated at 22 °C under 14-h day/10-h night cycle and 4000 lux light intensity using fluorescent lamps. After cultivation, the strains were transferred to a screw-capped test tube (15 mm × 150 mm) containing 9 mL of TS-15 medium as unialgal strains. A total of 61 strains were obtained. After incubation, the cells were collected by centrifugation for MC analysis (described above). The cells were extracted with 5% acetic acid (HOAc) and then with MeOH. The extracts were combined and applied to the InertSep RP-1 cartridge column (GL Science, Tokyo, Japan) pre-treated with MeOH and water. The cartridge was washed with 5% HOAc, water, and 20% MeOH. The MCs were eluted with 80% MeOH and the eluate was evaporated under reduced pressure.

### 4.7. Preparation of MC-FR and -WR

Cells of the *Microcystis* strain NIES-4344, producing MC-FR and -WR as major variants, were cultured in polycarbonate bottles containing 10 L of TS-15 medium at 25 °C under 12-h light/12-h dark cycle and 3000–5000 Lux light intensity using fluorescent lamps, with air-bubbling. Immediately after the confluent phase, cells were collected by continuous flow centrifugation (Suprema25; TOMY, Tokyo, Japan) at 9450× *g* for 30 min at a flow rate 10 L/60 min and then lyophilized. The lyophilized cyanobacterial sample (2.5 mg) was suspended in 300 mL of 5% HOAc, sonicated for 5 min, and then centrifuged at 2380× *g* for 10 min. The supernatant (5% HOAc extract) was collected. The precipitate was suspended in 300 mL of MeOH, sonicated for 5 min, and then centrifuged at 2380× *g* for 10 min. The supernatant (MeOH extract) was evaporated in a rotary evaporator to remove MeOH and combined with 5% HOAc extract. The combined extracts were mixed well, and the suspended mixture was centrifuged at 2380× *g* for 10 min. The supernatant was applied to the Inert Sep RP-1 cartridge column (GL science Inc., Tokyo, Japan) pre-treated with MeOH and water. The cartridge was washed with 5% HOAc, water, and 20% MeOH. The MCs were eluted with 80% MeOH and subjected to thin layer chromatography (TLC) and HPLC for further purification.

Structures of the isolated MCs were confirmed by extensive analyses of NMR and MS spectra. Concentrations of the isolated MCs were determined via quantitative NMR (qNMR) using caffeine as an internal standard. The NMR spectra were recorded using JNM-ECA500 spectrometer (JEOL Co.Ltd., Tokyo, Japan).

### 4.8. DNA Extraction, ftsZ Amplification and Next-Generation Sequencing

Phylogenetic analysis of the two *Microcystis* strains (NIES-4344 and NIES-4345) producing MC-FR and -WR variants was based on the *fts*Z genotype. DNA was extracted from 10 mL of the culture using Agencourt ChloroPure (BECKMAN COULTER, Fullerton, CA, USA). The amount of DNA was measured using the Qubit BR Assay Kit (Thermo Fisher Scientific Inc., MA, USA). The first PCR was conducted using a set of primers, ftsF and ftsR [17], with overhang sequences of Illumina, respectively. The second PCR was conducted using NEBNext Multiplex Oligos for Illumina. Amplification of the gene was confirmed by agarose gel electrophoresis, and each single band was purified using the QIAquick Gel Extraction Kit (Qiagen, Valencia, CA, USA). The gel-extracted samples were purified twice using Agencourt AMPure XP beads (Beckman Coulter, Brea, CA, USA). The quality of the samples was verified by Agilent 2200 TapeStation (Agilent Technologies, Inc., Santa Clara, CA, USA). DNA sequencing was performed using Illumina MiSeq (Illumina, San Diego, CA, USA) using the 600-cycle MiSeq Reagent Kit v3 (Illumina, San Diego, CA, USA).

### 4.9. Next-Generation Sequencing Data Analysis and Phylogenetic Analysis

Quality control of the sequencing data was conducted using CLC genomics workbench ver. 12.0 (Qiagen, Valencia, CA, USA), and OTU clustering was performed using the CLC Microbial genomics module ver.4.1 (Qiagen, Valencia, CA, USA), which was provided as a plugin for the CLC genomics workbench. DNA sequences of *fts*Z were aligned using MUSCLE [34] integrated into MEGA7 [35]. A maximum likelihood tree was constructed using MEGA7 [35] to identify the intraspecific groups within *Microcystis aeruginosa*.

## Figures and Tables

**Table 1:** PP2A inhibition assay and LC-MS analysis of sampling water.

Sampling Site No.	PP2A Inhibition (%), Mean ± SD (Test Solution)	MCs Identified by LC/MS (Crude Extract Solution)	Date of Collection	Sample Origin
1	43.27 ± 1.12	MC-RR, -LR, -LA	10/18/2008	Reservoir
2	7.03 ± 1.39	-	10/21/2008	Artificial pond
3	5.61 ± 1.43	-	10/21/2008	Artificial pond
4	3.49 ± 3.31	-	10/21/2008	Reservoir
5	5.66 ± 1.40	-	11/13/2008	Reservoir
6	9.24 ± 0.67	-	11/14/2008	Reservoir
7	82.37 ± 0.86	MC-RR, -LR, -FR, -WR	12/24/2008	Artificial pond
8	72.70 ± 0.94	MC-LR, -LA	12/24/2008	Reservoir
9	3.40 ± 3.12	-	12/24/2008	Artificial pond
10	80.50 ± 0.86	MC-RR, -LR	12/24/2008	Reservoir
11	78.71 ± 0.77	MC-RR, -LR	1/4/2009	Reservoir
12	71.33 ± 2.32	MC-RR, -LR	3/2/2009	Artificial pond
13	9.53 ± 0.29	-	10/21/2009	Reservoir

## Supplementary Materials

The following are available online at https://www.mdpi.com/2072-6651/11/9/521/s1, Figure S1: (**a**) Location of Okinawa prefecture and (**b**) collection sites of water sample. Figure S2: PP2A activity rate and calibration curve using net absorbance value obtained by the PP2A assay with MC-LR standard solution. Figure S3: Phylogenetic tree of the *ftsZ* Gene of *Microcystis aeruginosa*. The strain name “Oshiro-1-C5C5” and “Oshiro-2-A2” in Figure S1 show NIES-4344 and NIES-4345 respectively.
