# Supplementary material for: First Report of Microcystis Strains Producing MC-FR and -WR Toxins in Japan
Source: Toxins (Basel). 2019 Sep 9;11(9):521. doi: 10.3390/toxins11090521 (PMC6784158; doi:10.3390/toxins11090521)
Supplement: Supplementary file 1 [file toxins-11-00521-s001.zip › toxins-582041 supplementary/toxins-582041 supplementaryS1S2.pdf]

Supplementary Materials: First Report of *Microcystis* Strains Producing MC-FR and -WR Toxins in Japan

Tsuyoshi Ikehara, Kyoko Kuniyoshi, Haruyo Yamaguchi, Yuuhiko Tanabe, Tomoharu Sano, Masahiro Yoshimoto, Naomasa Oshiro, Shihoko Nakashima and Mina Yasumoto-Hirose

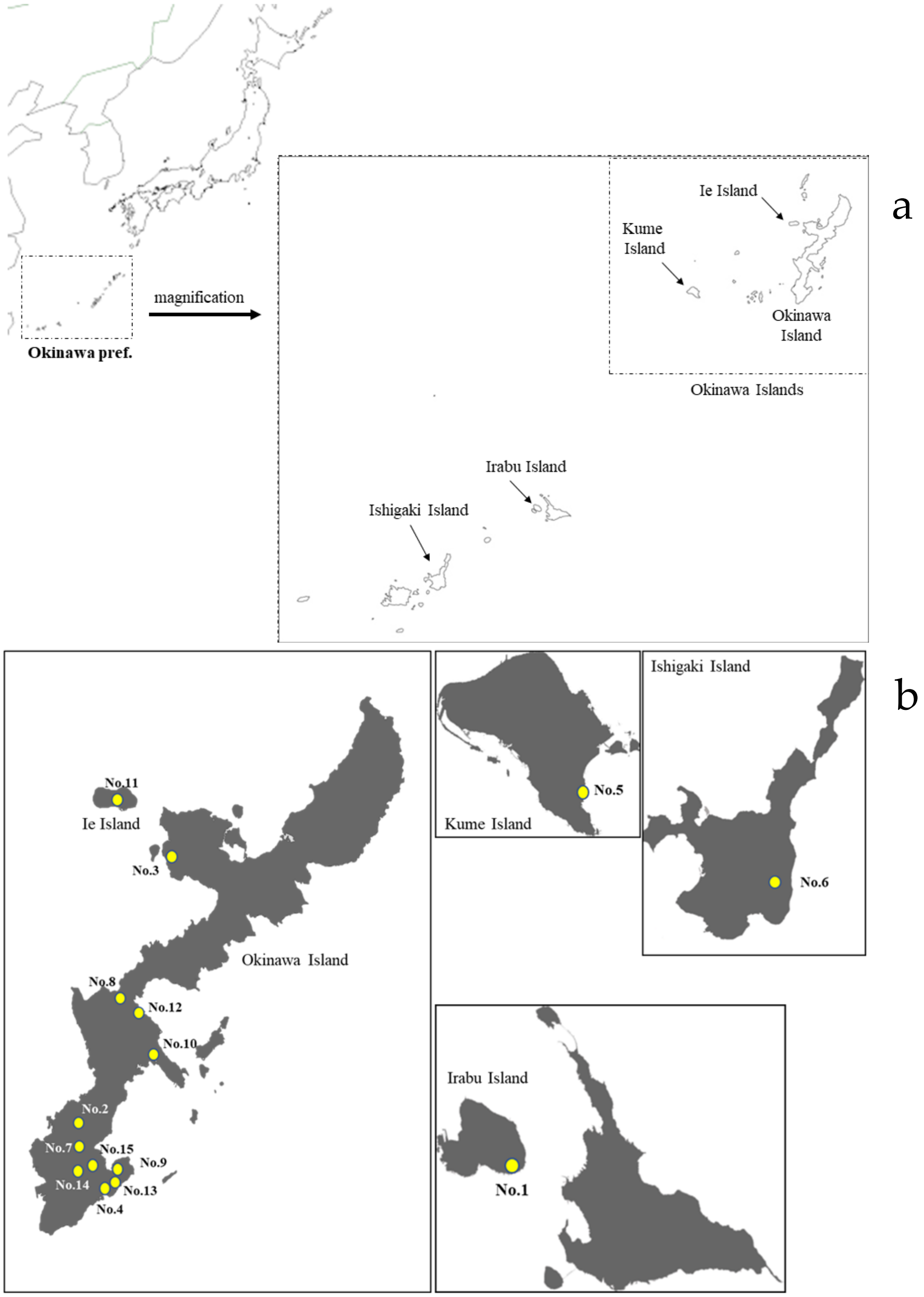

Figure 1. (**a**) Location of Okinawa prefecture and (**b**) collection sites of water sample.

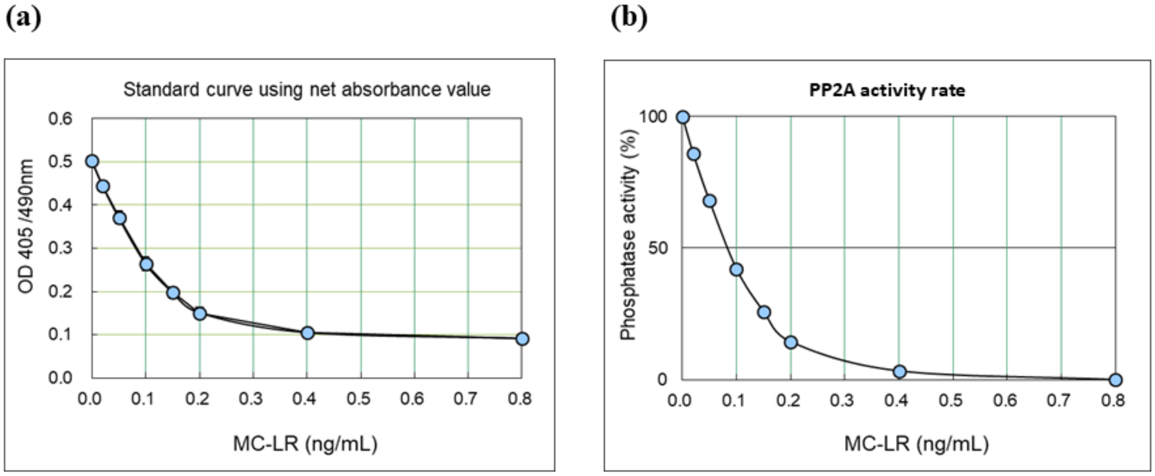

Figure 2. PP2A activity rate and calibration curve using net absorbance value obtained by the PP2A assay with MC-LR standard solution.
