# Supplementary figures and images for: First Report of Microcystis Strains Producing MC-FR and -WR Toxins in Japan
Source: Toxins (Basel). 2019 Sep 9;11(9):521. doi: 10.3390/toxins11090521 (PMC6784158; doi:10.3390/toxins11090521)

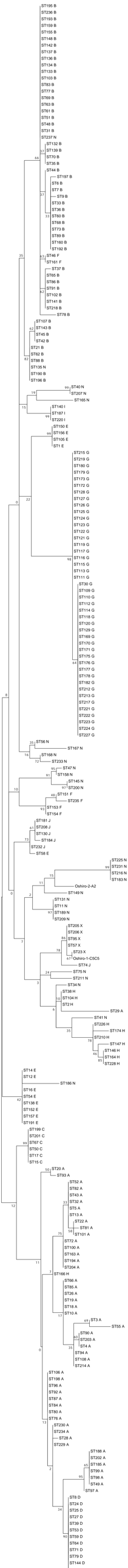

Supplement: Supplementary file 1 [file toxins-11-00521-s001.zip › toxins-582041 supplementary/toxins-582041 supplementaryS3.pdf]
